# Isotherm Modelling, Kinetic Study and Optimization of Batch Parameters Using Response Surface Methodology for Effective Removal of Cr(VI) Using Fungal Biomass

**DOI:** 10.1371/journal.pone.0116884

**Published:** 2015-03-18

**Authors:** Melvin Samuel S, Evy Alice Abigail M, Ramalingam Chidambaram

**Affiliations:** School of Bioscience and Technology, VIT University, Vellore, Tamil Nadu, India; University of Illinois at Urbana-Champaign, UNITED STATES

## Abstract

Biosorption is a promising alternative method to replace the existing conventional technique for Cr(VI) removal from the industrial effluent. In the present experimental design, the removal of Cr(VI) from the aqueous solution was studied by *Aspergillus niger* MSR4 under different environmental conditions in the batch systems. The optimum conditions of biosorption were determined by investigating pH (2.0) and temperature (27°C). The effects of parameters such as biomass dosage (g/L), initial Cr(VI) concentration (mg/L) and contact time (min) on Cr(VI) biosorption were analyzed using a three parameter Box–Behnken design (BBD). The experimental data well fitted to the Langmuir isotherm, in comparison to the other isotherm models tested. The results of the D-R isotherm model suggested that a chemical ion-exchange mechanism was involved in the biosorption process. The biosorption process followed the pseudo-second-order kinetic model, which indicates that the rate limiting step is chemisorption process. Fourier transform infrared (FT-IR) spectroscopic studies revealed the possible involvement of functional groups, such as hydroxyl, carboxyl, amino and carbonyl group in the biosorption process. The thermodynamic parameters for Cr(VI) biosorption were also calculated, and the negative ∆Gº values indicated the spontaneous nature of biosorption process.

## Introduction

Increase in industrialization and anthropogenic activities have emerged as a major problem in recent years due to the release of large amounts of heavy metals as waste directly into the surface waters, ponds and rivers. These heavy metals disturb the eco-system and make it unfit for human consumption [1]. Once released into the environment, they accumulate into living tissues via the food chain and cause toxicity even at lower concentrations. Chromium is one of the heaviest metals, and it is most hazardous in nature [2]. The effluents of textile, tannery, electroplating, metal finishing, metallurgical, leather tanning, dye, wood preservation and battery manufacturing-industries were found to contain Cr(VI) [3]. According to the U.S. Environmental Protection Agency, the maximum permissible limit of Cr(VI) in natural water is only 0.05 mg/L [4].

Cr(VI) toxicity is essentially based on its negatively charged ions, which forms complexes. The formed complexes easily penetrate the cellular membranes of living cells through sulfate ionic channels. Once it enters the cell membrane, it readily undergoes a reduction reaction which leads to the formation of serious harmful reactive intermediates [5]. At lower concentrations, Cr(VI) is known to cause respiratory tract disorders, allergies and eczema, whereas at higher concentrations it leads to colon cancer, digestive tract cancer and lung cancer [6–10]. Therefore, it is vital to remove Cr(VI) from industrial effluents before discharging it into the environment.

In the recent years, Cr(VI) removal from the contaminated site has drawn the attention of researchers due to its toxic properties. The conventional techniques widely used for Cr(VI) removal are electrochemical treatment, chemical precipitation, membrane process, reverse osmosis, ion exchange, liquid extraction, electro dialysis, evaporation and sorption methods. However, the above mentioned methods are quite inefficient due to high operational costs and investment, risk of secondary pollution, expensive treatment and disposal of generated secondary pollutants, which are not eco-friendly [3, 11].

Commercial adsorbents, such as activated carbon were also used for Cr(VI) removal. Although activated carbon is efficient, the process was found to be quite expensive. The addition of chelating agents for removing inorganic molecules makes the process much more complex and costly. Thus, there is a necessity to develop a process by which Cr(VI) can be removed effectively and economically from the contaminated site. Therefore, at present, an emphasis is given to adsorbents from a biological origin to remove and recover Cr(VI) even at relatively low concentrations (10–100 mg/L), which is impossible to be removed by other methods [2,12–13].

There are several reports on biosorption of Cr(VI) by various strains of bacteria, algae, fungi, and seaweed [14–17]. Fungi seems to be the most efficient in Cr(VI) removal, mainly because of its adaptability to stress conditions, fast growing nature and minimal nutrient requirement. Fungi can detoxify heavy metals using mechanisms such as ion exchange, adsorption, extra cellular precipitation, intracellular precipitation and valence transformation. The fungal cell wall is composed of chitin, which is a long linear chain of beta-1,4-linked N-acetylglucosamine and also possesses proteins, glucan, polymers and functional groups such as carboxyl, phosphoryl, hydroxyl, amino and imidazole on their surface. This aids in the attachment of Cr(VI) onto the surface of biosorbent by valence forces i.e., through the ion exchange of electrons and effective removal of Cr(VI) [11].

The aim of the present experimental study was (a) to isolate a fungal biomass with high Cr(VI) removal capacity from chromium contaminated soil near the industrial effluent area, (b) to determine the effects of important experimental variables such as initial Cr(VI) concentration, contact time and biomass dosage on Cr(VI) percentage removal using a three-variable Box-Behnken Design (BBD), (c) to find the rate of biosorption mechanism through isotherm and kinetic models, (iv) to study the effect of temperature on biosorption through thermodynamic parameters and (v) to characterize the biosorbent before and after biosorption *via* the FT-IR analysis. Therefore, this present work focuses on the aspects of chromium(VI) biosorption by the fungal strain *Aspergillus niger* MSR4 biomass.

## Materials and Methods

### Chemicals and reagents

Analytical grade potassium dichromate (K_2_Cr_2_O_7_) was purchased from Sigma Aldrich, Bangalore, India. A stock solution (1000 mg/L) of Cr(VI) was prepared by dissolving exact quantities of K_2_Cr_2_O_7_ in deionized-distilled water. All the other chemicals used in the study were of analytical grade.

### Isolation of chromium resistant fungi

A fungus was isolated from the contaminated soil of an industrial effluent area in Vellore, Tamil Nadu, India and was designated as the MSR4 strain. No specific permission was required for sample collection from the above mentioned site located at 12.9°N and 79.9°E. The culture was maintained on thepotato dextrose agar (PDA) medium slants at 4°C throughout this study. For molecular identification, the fungal genomic DNA was isolated using the Insta Gene TM matrix genomic DNA isolation kit and the 18s rRNITS region was amplified using universal primers “ITS1”-“TCCGTAGGTGAACCTGCGG” and “ITS4”-”TCCTCCGCTTATTGATATGC“. The sequencing regions were submitted to the GenBank under the accession number KJ881377.

### Preparation of biosorbent

The fungi were cultured in a filamentous form under the aerobic condition for 3 days in a yeast extract peptone glucose (YPG) media, which consists of yeast extract of 3 g/L, peptone 10 g/L and dextrose (a-D-glucose) 20 g/L. The pH of the growth media was adjusted to 4.5 with 0.1M HCl. After three days of incubation, the biomass was collected and dried at 60°C temperature in an oven for 24 hours. The dried biomass was sieved through a 150-mesh sieve and used for biosorption experiments.

### Batch biosorption experiments

Batch experiments, designed by RSM (Response surface methodology), were conducted in 250 ml Erlenmeyer flasks at pH 2.0 and 27°C in order to study the effect of biomass dosage, initial Cr(VI) concentration and contact time, which could improve the removal of Cr(VI) onto the MSR4 biomass from the solution. Biosorption studies were performed by varying the biomass dosage (1–3 g/L), Cr(VI) concentration (25–100 mg/L) and contact time (15–60 min). The highest and l ower limits of the independent variables are shown in Table 1.

**Table 1:** Experimental ranges and levels in the experimental design.

Factors	Range and level		
	-1	0	+1
_X_1: Biomass dosage (g/L)	1	2	3
_X_2: Initial Cr(VI) concentration	25	67.5	100
_X_3: Contact time (min)	15	37.5	60

For each study, the control flask (without biomass) was also maintained. The experiments were carried out in triplicates, and the mean average value was used for the analysis. After biosorption, the solution was filtered through a Whatmann filter paper no. 1, and the Cr(VI) concentration was analysed by Atomic Absorption spectroscopy (AAS). The differences in Cr(VI) concentration before and after the biosorption were calculated to find out the percentage of hexavalent chromium adsorbed by the biomass. All the experimental data was performed and analyzed using Design Expert software (Version 9.0, stat-Ease, Inc., Minneapolis, United States).

### Box-Behnken experimental design

Box-Behnken Design (BBD) was used for the experimental design. It is a well suited model for fitting a quadratic surface and also works best for the optimization process [18]. To evaluate the influence of operating parameters on Cr(VI) biosorption by MSR4 biomass, the three independent variables *viz*. biomass dosage (X_1_), initial Cr(VI) concentration (X_2_) and contact time (X_3_) were chosen. A total of 17 experiments were designed using the formulae:

$$N=k^{2}+k+cp$$

Where k is the factor number and cp is the replicate number of the central point [19]. The second order polynomial equation was used to correlate the dependent and independent variables

$$Y=\;b_{o\;}\;+\;b_{1}x_{1}+b_{2}x_{2}+b_{3}x_{3}+b_{12}x_{1}x_{2}+b_{13}x_{1}x_{3}+b_{23}x_{2}x_{3}+b_{11}x_{1}^{2}+\;b_{22}x_{2}^{2}+b_{33}x_{3}^{2}$$ (1)

Where Y is a response variable of removal efficiency, b_0_ is constant, b_1_, b_2_ and b_3_ are linear coefficients, b_12_, b_13_ and b_23_ are cross product coefficients, b_11_, b_22,_ b_33_ are quadratic coefficients; x_i_ is coded experimental levels of the variables, biomass dosage (_X_1), initial Cr(VI) concentration (_X_2) and contact time (_X_3). The optimum values of these factors were obtained by solving the regression equation as well as by analysing the response surface plots. Preliminary experiments were performed to determine the extreme values of the variables.

### Equilibrium adsorption isotherms

To evaluate the equilibrium data for biosorption of Cr(VI) onto MSR4 biomass, the isotherm models such as Langmuir, Freundlich, Dubinin-Radushkevich (D-R), Temkin, Harkins and Hasley were employed. The non-lineralized equations of the above mentioned isotherms are tabulated in Table 2.

**Table 2:** List of isotherm model equations used in the study.

Isotherms	Non- Linearized form	References
**Langmiur**	$q_{e}=\;\frac{q_{\max}bC_{eq}}{1+bC_{eq}}\;$	[20]
**Freundlich**	$q_{e}=K_{f}C_{eq}{}^{1/n}$	[21]
**Dubinin-Radushkevich (D-R)**	$q_{e}=q_{\max}e_{}{}^{-\beta \varepsilon^{2}}$	[22]
**Temkin**	$q_{e}=\;\frac{RT}{b_{T}}\;(\ln\;A_{T\;}C_{eq})$	[23]
**Harkins**	-	[24]
**Hasley**	$q_{e}=\;(\frac{K_{H}}{C_{e}}){\;}^{\frac{1}{\eta H}}$	[25]

### Biosorption kinetics

Several kinetic models were used in the past decade in order to describe the mechanism of the biosorption process. In this study, kinetic models such as fractional power, zero-order, first order, pseudo-first order, Elovich, second order, pseudo-second order and intraparticle diffusion kinetics, were used to fit the biosorption data for Cr(VI) removal by the MSR4 strain. The equations of the kinetic models were utilized to investigate the mechanism, and the rate controlling step of the biosorption process is tabulated in Table 3.

**Table 3:** Summarized the mathematical equations of the mentioned kinetics models.

Kinetic model	Linearized form	References
**Fractional power**	ln*q*_*t*_ = ln*k* + *v* ln*t*	[26]
**Zero order**	(*q*_*t*_—*q*_*e*_) = *q*_*e*_—*K*_o_ *t*	[27]
**First oder**	ln(*q*_*t*_—*q*_*e*_) = ln*q*_*e*_—*K*_1_ *t*	[28]
**Pseudo- first order**	$\log\;(q_{e}\;-\;q_{t})=\log q_{eq}-\;\frac{K_{1}t}{2.303}\;$	[29]
**Second order**	$\frac{1}{\left(q_{t}\;-\;q_{e}\right)}=\;\frac{1}{q_{e}}+\;K_{2}t$	[30]
**Pseudo—second order**	$\frac{t}{q_{t}}\;=\;\frac{1}{K_{2}q_{e}^{2}{}_{}}\;+\;\frac{1}{q_{e}}\;t$	[31]
**Elovich**	$q_{t\;}=\;\frac{1}{b_{e}}(\ln\;a_{e}b_{e})+\frac{1}{b_{e}}\ln t$	[32]
**Intraparticle diffusion**	log*q*_*t*_ = log*k*_*id*_ + 0.5log*t*	[33]

### Thermodynamic study

To investigate the thermodynamic nature of MSR4 biosorbent for Cr(VI) biosorption, different parameters *viz*. ∆G°(Gibbs free energy), ∆H° (enthalpy) and ∆S° (entrophy) were calculated using the equations

$$\Delta G{}^{\circ}=\;-RT\;\ln K_{c}$$ (2)

ΔG°=  ΔH°−T ΔS° (3)

$$K_{c}=\frac{q_{e}}{c_{f}}$$ (4)

Where K_c_ is the distribution coefficient for adsorption, R is the gas constant (KJ/mol/K) and T is the absolute temperature (Kelvin). Based on the Van’t Hoff plot of ln Kc verses 1/T, the values of ∆H^o^ and ∆S^o^ were determined from the slope and intercept.

### Fourier transform infrared spectroscopy (FT-IR)

The FT-IR analyses within the range of 400–4000 cm^-1^ were recorded with a IR spectrometer (IR Affinity-1, Shimadzu, Japan) for Cr(VI) biosorption by MSR4 biomass at the contact time of 60 min.

### Desorption and regeneration studies

Desorption studies were performed using the eluents *viz*. 0.1 M HNO_3_, 0.1 M HCl and 0.1 M NaOH. After Cr(VI) biosorption, the loaded-MSR4 biomass were placed in an eluent solution and shaken at 120 rpm at 27°C. The desorbed chromium in the supernatant was taken for analysis using AAS.

## Results and Discussion

### Isolation and identification of Cr(VI) resistant fungal strain MSR4

**Fig 1:**
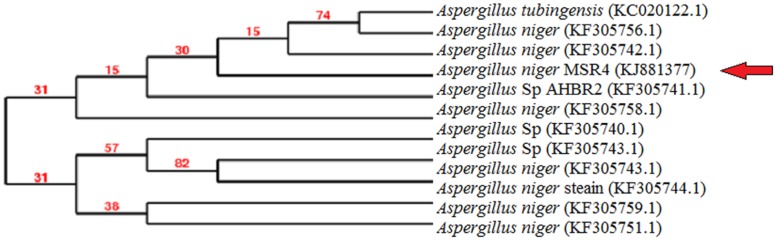
Phylogenetic tree of Aspergillus niger MSR4 strain based on the nucleotide sequences of the partial 18S rRNA genes. The tree was constructed using neighbour joining method.

The fungal strain MSR4 was isolated from hexavalent chromium contaminated soil and was identified through molecular level characterization. The 18S rRNA gene sequence analysis of the isolated strain exhibited 99% homology with *Aspergillus niger* (GenBank accession number: KF305751.1) and *Aspergillus niger* (GenBank accession number: KF3057421). Therefore, the strain MSR4 was designated as *Aspergillus niger* MSR4. The sequencing results were submitted in the NCBI GenBank database (Accession number: KJ881377) and a phylogenetic tree (Fig. 1) was constructed for the strain MSR4, using TREEVIEW software (version 1.6.6).

### BBD model validation for Cr(VI) biosorption

The present study of the biosorption percentage for Cr(VI) was investigated using a response surface methodology according to the Box-Behnken model which consisted of 17 experiments. In order to achieve optimal biosorption percentage, the effect of three operating variables *viz*. biosorbent dosage, initial Cr(VI) concentration and contact time were selected. The batch experiments were conducted using BBD to visualize the effects of independent variables on the response at optimal pH (2.0) and temperature (27°C) [34]. The statistical significance of the second order polynomial model given empirical relationship is coded in units to find out the relationship between variables and response as follows

$$\begin{array}{l} Y=\;+35.78\;+2.6\;4\;x_{1}+\;3.41\;x_{2}+\;24.432\;x_{3}-\;0.522\;x_{1}x_{2}\;+\;0.25\;x_{1}x_{3}+\;2.14\;x_{2}x_{3}\;-4.77\;x_{1}^{2}\\ -1.70\;x_{2}^{2}-0.92\;x_{3}^{2} \end{array}$$

Where, Y is the response of the biosorption percent of Cr(VI), x_1_, x_2_ and x_3_ corresponds to independent variables of biosorbent dosage (g/L), initial Cr(VI) concentration (mg/L) and contact time (min), respectively (Table 4).

**Table 4:** Box-Behnken Design matrix for three factors along with observed response for Cr(VI) biosorption by MSR4 biosorbent.

Run	A:Biomass dosage (g/L)	B:Intial Cr(VI) concentration (mg/L)	C:Contact time (min)	R1-Cr(VI) percentage removal (%)
1	1.00	-1.00	0.00	30.89
2	-1.00	0.00	-1.00	5.89
3	0.00	1.00	-1.00	9.24
4	0.00	0.00	0.00	35.78
5	1.00	0.00	1.00	54.8
6	0.00	1.00	1.00	63.82
7	1.00	1.00	0.00	36.78
8	0.00	-1.00	-1.00	6.78
9	-1.00	1.00	0.00	28.78
10	0.00	0.00	0.00	35.78
11	0.00	0.00	0.00	35.78
12	1.00	0.00	-1.00	6.89
13	-1.00	-1.00	0.00	20.8
14	0.00	-1.00	1.00	52.8
15	-1.00	0.00	1.00	52.78
16	0.00	0.00	0.00	35.78
17	0.00	0.00	0.00	35.78

The model F-value (120.68) indicated that the quadratic model is significant. There was only 0.01% chance that the F-value could occur due to noise. “Adequate Precision" measures the signal to noise ratio, and a ratio greater than 4 is desirable. The ratio of 36.161 indicates an adequate signal, and the model can be used to navigate the design space. Values of "Prob > F" less than 0.0500 indicate model terms are significant. In this design, x_1_, x_2_, x_3_, x_1_^2^ are significant model terms. Using ANOVA, the regression coefficient of the predicted versus experimental values (R^2^ = 0.98 and adj-R^2^ = 0.87) were also calculated (Table 5). The high R^2^ showed that the predicted responses fit the biosorption percent of MSR4 strain for the Cr(VI) biosorption.

**Table 5:** ANOVA analysis for response surface second order model in relation to Cr(VI) biosorption.

Source	Sum of Squares	df	Mean Square	F Value	p-value Prob > F	
Model	5060.27	9	562.25	120.68	< 0.0001	Significant
A-A-Biomass dosage	55.70	1	55.70	11.96	0.0106	
B-B-Intial Cr(VI) concentration	93.50	1	93.50	20.07	0.0029	
C-C-Contact time	4772.64	1	4772.64	1024.42	< 0.0001	
AB	1.09	1	1.09	0.23	0.6431	
AC	0.26	1	0.26	0.056	0.8200	
BC	18.32	1	18.32	3.93	0.0878	
A^2	95.75	1	95.75	20.55	0.0027	
B^2	12.15	1	12.15	2.61	0.1504	
C^2	3.57	1	3.57	0.77	0.4102	
Residual	32.61	7	4.66			
Lack of Fit	32.61	3	10.87			
Pure Error	0.000	4	0.000			
Cor Total	5092.88	16				

**Fig 2:**
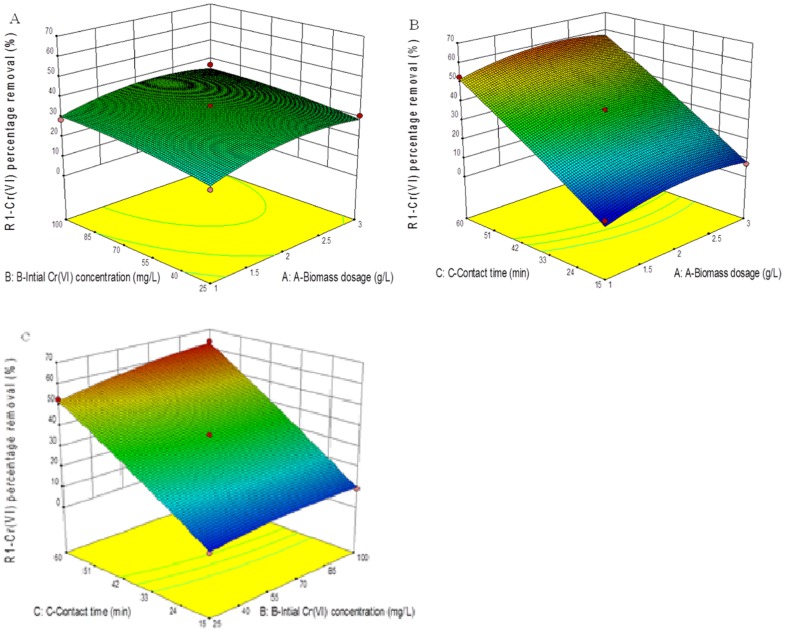
Respone surface plots showing the combined effects of (A) biomass dosage and initial Cr(VI) concentration (B) initial Cr(VI) concentration and contact time (C) contact time and biomass dosage on the removal (%) of Cr(VI).

Fig. 2a shows the 3D plot, which depicts the simultaneous effect of biomass dosage and initial Cr(VI) concentration in the aqueous solution on Cr(VI) removal efficiency, when contact time was kept constant. The removal efficiency of Cr(VI) increased in proportion to the increase of biomass dosage at a lower biomass dosage. In the range of Cr(VI) concentrations, the percentage removal of Cr(VI) initially rose from 25.01 to 37.65% and then gradually decreased. At the initial concentration of 62.5 mg/L, the removal efficiency was enhanced, but reduced when the biomass dosage rose to above 2 g/L.

**Fig 3:**
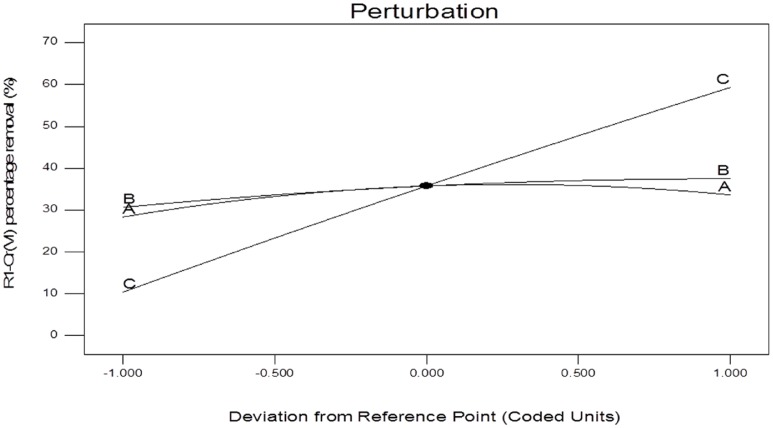
Overlay plot of perturbation of the three variables tested.

Similarly in the Fig. 2b, the variation of biomass dosage with contact time is illustrated. As it can be seen, the percentage removal increased from 8.03 to 56.96%, when the initial Cr(VI) concentration was kept at central level of 62.5. Finally, the 3D response surface plot reveals the influence of the interaction between initial Cr(VI) concentration and contact time. The removal efficiency increased at higher levels of contact time. From Fig. 2a, b and c, maximum Cr(VI) removal percentage (63.82%) was achieved at a biomass dosage of 2 g/L and initial concentration of 62.5 mg/L, while the contact time was 37.5 min. In Fig. 3 response design, the perturbation plot for each factor changes or moves from the reference point. In the model, the factor C center point, (Contact time) shows a high response effect on as it changes from the reference point. Higher the contact time, greater the removal percentage (%) of the Cr(VI) ion. Factor A (Biomass dosage) and B (chromium concentration) has the same effect as factor C. Additionally the biosorption capacity obtained in the present study was compared with other data reported in the literature (Table 6)

**Table 6:** Comparison of other fungal biosorbents from literature with the present work.

Name of fungi	Sorption capacity (mg/g)	Reference
*Aspergillus niger*	30.1	[35]
*Aspergillus flavus*	0.335	[36]
*Coriolus versicolor*	44.25	[37]
*Lentinus sajor-caju* (free)	23.32	[38]
*Mucor hiemali*	53.5	[39]
*Penicillium purpurogenum*	40	[40]
*Rhizopus arrhizus*	23.88	[41]
*Saccharomyces cerevisiae*	32.6	[42]
*Penicillium purpurogenum*	40	[40]
***Aspergillus niger* MSR4**	**71.9**	**Present study**

### Equilibrium isotherm studies for Cr(VI) biosorption

**Fig 4:**
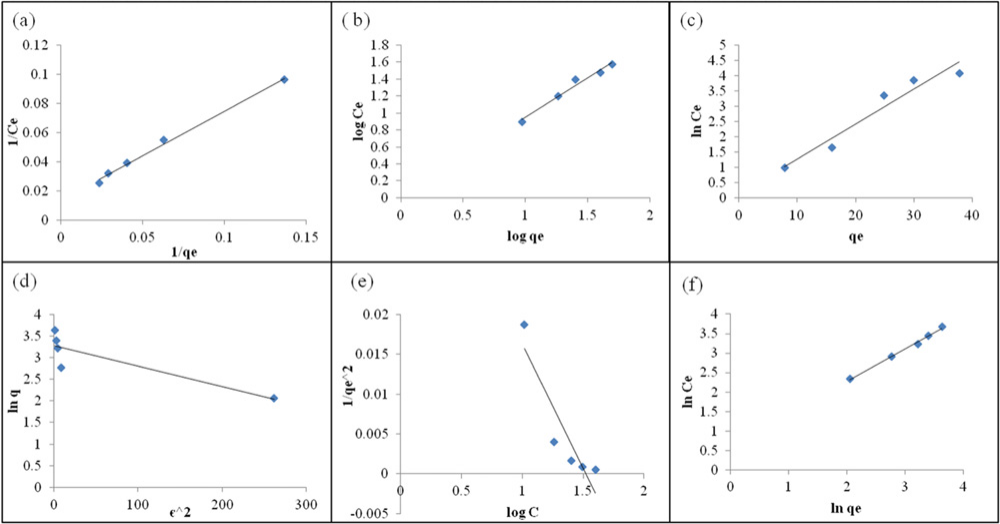
a) Langmuir isotherm b) Freundlich isotherm c) Tempkin isotherm d) D-R isotherm e) Harkin-Jura isotherm and f) Hasley isotherm for Cr(VI) biosorption by MSR4 strain (initial Cr(VI) concentration: 25–100 mg/L, biomass dosage: 2g/L, pH: 2.0, temperature: 27°C.

Equilibrium sorption isotherm is described by constants whose values express the surface properties and affinity of the adsorbent sorption equilibrium which is established when the concentration of sorbate in the bulk solution is in dynamic balance with that at the adsorbent surface. To quantify the affinity of MSR4 biomass for Cr(VI) ions, isotherm models such as Langmuir, Freundlich, D-R, Temkin, Harkins-Jura and Hasley isotherm, were used to analyse the data obtained from the biosorption process. The graphical representation of all isotherm models are given in Fig. 4.

It could be seen from Table 6 that the equilibrium study gave a good fit for the Langmuir model with high regression coefficient (>0.99), in comparison to the other tested isotherm models. This confirms the surface homogeneity of the adsorbent. The Freundlich isotherm model is usually applied for non-ideal reversible adsorption processes and it gives the information regarding multilayer adsorption with the non-uniform distribution of heat which takes place on the heterogeneous surface [21]. In the present study, a low regression coefficient (R^2^) less than 0.99, suggests that the mode of adsorption is not heterogeneous in nature. When the D-R model was applied to check whether the Cr(VI) biosorption by MSR4 biosorbent follows the physisorption or chemisorption process [22], the obtained value of mean free energy (E) was found to be greater than 8 KJ/mol. The E value corresponds with the mechanism of Cr(VI) biosorption onto the MSR4 biosorbent, which is a chemical-ion exchange process (Table 7).

**Table 7:** Equilibrium isotherm constants of Cr(VI) biosorption on MSR4 biomass.

Isotherms	
**Langmiur**	**Temkin**
q_max_ (mg/g)– 71.9	A (J/mol)- 22.7
B—0.031	B (L/g)– 0.11
R_L_—0.24	R^2^—0.93
R^2^—0.999	
**Freundlich**	**Harkins-Jura**
K_f_—1.058	A—22.7
N—1.071	B—1.57
R^2^_–_0.967	R^2^–0.89
**Dubinin-Radushkevich(D-R)**	**Hasley**
E(KJ/mol)– 8.96	n—0.78
q_max_(mg/g)– 27.08	R^2^–0.96
ß—0.006	
R^2^—0.744	

The Temkin isotherm model gives the information regarding the adsorbent-adsorbate interaction. It is based on the assumption that the free energy of sorption is a function of surface coverage [23]. The low R^2^ value, obtained from the model, implied that the heat of the adsorption of all Cr(VI) molecules in a layer did not decrease with the surface coverage of the adsorbate-sorbate interaction. In order to determine whether multilayer adsorption has taken place or not, Harkins-Jura and Hasley isotherm equations were employed. The Harkins-Jura model also explains the existence of heterogeneous pore distribution [24]. The low R^2^ value obtained from the models exhibited that the adsorption of Cr (VI) onto the MSR4 strain, which did not follow a multilayer adsorption mode. Therefore, it was evident from the present study that the Cr(VI) molecules were adsorbed onto the surface of MSR4 biosorbent following the monolayer adsorption mode and does not permit transmigration of the adsorbate in the plane of the surface [20]. It also implies that each Cr(VI) molecule owns its enthalpy and activation energy at the same time.

### Kinetic modelling for Cr(VI) biosorption

The applicability of kinetic models *viz*. fractional power, zero order, first order, pseudo first order, Elovich, pseudo second order and intraparticle diffusion kinetics were investigated by measuring the regression coefficients. The plot of pseudo second order model (data not shown) for biosorption of Cr(VI) by MSR4 biomass showed that experimental data fitted well with the pseudo second order model (R^2^>0.99). In case of the other models such as fractional power, zero order, first order, pseudo first order and Elovich model, the R^2^ values were found to be very low. Significant differences were observed between calculated and experimental uptake values (Table 8). So, the best fitted pseudo second order models explained the mechanism of the Cr(VI) biosorption onto the MSR4 biosorbent to be chemisorption process.

**Table 8:** The values of the parameters for different kinetic models fitted to the Cr(VI) biosorption kinetics on MSR4 biomass at various concentrations.

Concentration (mg/L)	25	50	75	100	125
**Experimental q_e_**	6.45	14.18	22.22	31.91	32.79
**Fractional power**					
**K**	1.935	5.98	10.07	11.45	13.45
**V**	0.381	0.25	0.27	0.29	0.30
**R** ^**2**^	0.87	0.89	0.89	0.90	0.98
**Zero order**					
**K**_**0**_	0.142	0.312	0.366	0.544	0.546
**q**_**e**_	3.91	8.45	2.26	2.79	2.46
**R** ^**2**^	0.86	0.87	0.87	0.88	0.89
**First order**					
**K**_**1**_	0.165	0.11	0.076	0.066	0.051
**q**_**e**_	4.20	8.18	16.11	27.93	22.64
**R** ^**2**^	0.82	0.84	0.87	0.88	0.89
**Pseudo- first order**					
**K**_**1p**_	0.16	0.11	0.076	0.051	0.066
**q**_**e**_	4.21	8.19	16.18	22.78	28.11
**R** ^**2**^	0.97	0.98	0.97	0.97	0.92
**Elovich**					
**a**_**e**_	5.62	28.73	29.12	31.53	28.21
**b**_**e**_	0.713	0.412	0.267	0.186	0.155
**R** ^**2**^	0.984	0.989	0.968	0.963	0.964
**Second order**					
**K**_**2**_	0.174	0.036	0.020	0.019	0.008
**q**_**e**_	4.21	8.19	16.12	18.34	22.34
**R** ^**2**^	0.81	0.82	0.85	0.87	0.89
**Pseudo—second order**					
**K**_**2p**_	3.38	1.678	0.366	0.209	0.198
**q**_**e**_	6.450.998	14.18	22.83	33.110	34.480
**R** ^**2**^	0.998	0.995	0.998	0.997	0.993
**Intraparticle diffusion**					
**K**_**id**_	0.94	1.78	3.53	5.38	5.52
**C**	2.62	6.87	7.16	7.61	8.01
**R** ^**2**^	0.74	0.80	0.82	0.90	0.92

The intraparticle diffusion model was plotted between a solute adsorbed against the square root of contact time to check the effect of mass transfer resistance on the binding Cr(VI) ions to the biosorbent MSR4. The low R^2^ value and non-linear plot showed that the intraparticle is not responsible for the biosorption kinetic of Cr(VI) onto the biosorbent in this experiment. The plots also exhibited multi-linearity, thus indicating three biosorption steps i.e., initial curved portions that attribute to instantaneous biosorption stage followed by the gradual sorption stage where intraparticle diffusion was rate controlled, followed by the final equilibrium stage due to a low solute concentration. These results indicated that intraparticle diffusion is not the rate limiting step.

### Thermodynamic study for Cr(VI) biosorption

The thermodynamic parameters are tabulated in Table 9. The negative values of ∆G° indicated the thermodynamically feasible nature and spontaneity of the process. The negative ∆G° was found to increase due to any increase in temperature. This shows an increase in the feasibility of Cr(VI) biosorption at higher temperatures, which might be due to the fact that higher temperatures cause the diffusion of Cr(VI) molecules from the solution to the biosorbents to be faster. This is also due to the increase in solubility of Cr(VI) ions. The negative ∆H° indicates the exothermic nature of the process at 22–42°C whereas the positive value of ∆S° reveals the increased randomness at the solution-solid interface during the Cr(VI) biosorption onto the fungal biomass, MSR4.

**Table 9:** Thermodynamics parameters for Cr(VI) biosorption on fungal biomass MSR4.

Temperature (K)	∆G^o^ (kJ/mol)	∆H^o^ (kJ/mol)	∆S^o^ (J/mol-K)
295	6.382	170.78	557.95
300	-35.48	-	-
305	-10.71	-	-
310	24.22	-	-

### FTIR analysis of Cr(VI)-biomass interaction

**Fig 5:**
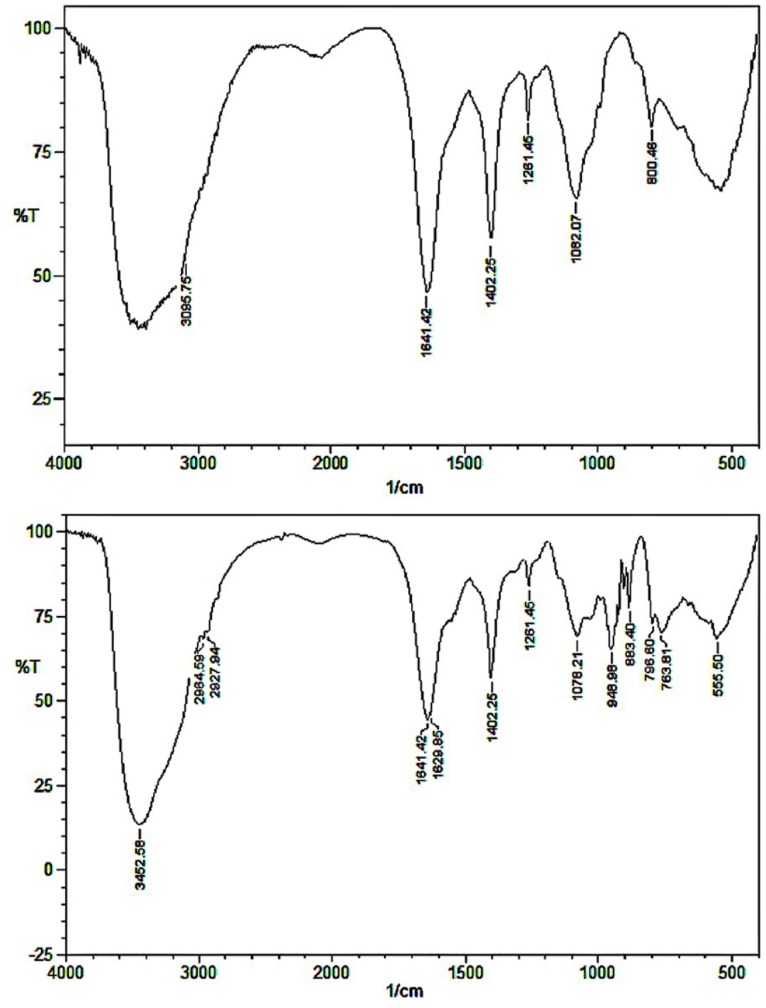
The IR maximum of absorption for different functional groups, before and after Cr(VI) sorption are presented.

Fig. 5, summarized the IR maximum of absorption for different functional groups, before and after Cr(VI) sorption are presented. The obtained spectrum showed a significant difference merely based on shifts in some peaks. The peak shift from 3095 cm^-1^ to 3452 cm^-1^ denotes the attachment of Cr(VI) on the-OH and-NH group. The peaks at 1641 cm^-1^ and 1402 cm^-1^ remains same before and after biosorption. The peak at 2964 cm^-1^ and 2827 cm^-1^ in the adsorbed biomass denotes the C–H/CO stretching vibration. The peak shift at 1641–1629 cm^-1^, 1281–1261 cm^-1^ and 1082–1078 cm^-1^ revealed changes in the stretching frequency of carboxyl group upon binding of the Cr(VI) molecule. A broad and moderately intense peak was visible at the range of 798–763 cm^-1^, representing Cr-O vibrations, Moreover, a peak shift from 800–883 cm^-1^ was also observed after interaction with Cr(VI).

### Desorption studies

Desorption studies were carried out for the fungal biomass MSR4 by using various reagents such as 0.1M HNO_3_, 0.1M HCl and 0.1M EDTA. From the results it is evident, that with increase of HCl, HNO_3_ concentration, the desorption rate also increased at the initial and then reaches equilibrium. There was a gradual decrease in Cr(VI) sorption with an increase in the number of cycles. After a sequence of five cycles, the Cr(VI) uptake capacity of the sorbent was reduced from 88.01% to 72%. The lost in the sorption capacity of the biomass for metal ions was found to be 16%. This might be due to the ignorable amount of biomass lost during the sorption–desorption process. It was observed that the Cr(VI) ions could be desorbed up to a maximum of 88% which could be useful in industrial purposes. Also, it was found that MSR4 biomass can be desorbed up to a maximum of HNO_3_, followed by HCl (78.9%) and EDTA (72.8%). These results indicate that the MSR4 biomass could be used repeatedly in Cr(VI) sorption studies.

## Conclusion

The fungus, *Aspergillus niger* MSR4, isolated from Cr(VI) contaminated soil was efficiently used for the biosorptive removal of Cr(VI) from an aqueous solution. The effect of biomass dosage, Cr(VI) initial concentration and contact time was studied using a Box-Behnken design for the optimum removal of Cr(VI) by the MSR4 strain. Among the equilibrium models tested, Langmuir isotherm model was found to be the best fit for the obtained experimental data, thus suggesting a monolayer biosorption mode. The pseudo-second order kinetic model fitted best among the other kinetic models tested, thereby describing the mechanism of Cr(VI) biosorption as a chemisorption process. FT-IR analysis confirmed the role of surface functional groups present on the fungal biomass in the biosorption process. Thus, the present study portrays the use of MSR4 fungal biomass as an alternative method for treatment of Cr(VI), instead of the currently available conventional methods.
